# Incidental findings on non-contrast abdominal computed tomography in an asymptomatic population: Prevalence, economic and health implications

**DOI:** 10.1371/journal.pone.0328049

**Published:** 2025-08-04

**Authors:** Alexander Ritter, Maximilian Sabev, Olivier Bonny, Gregoire Wuerzner, Thomas Ernandez, Florian Buchkremer, Stephan Segerer, Daniel G. Fuster, Beat Roth, Nilufar Mohebbi, Lena Jellestad, Andreas M. Hoetker, Carsten A. Wagner, Harald Seeger

**Affiliations:** 1 Division of Nephrology, University Hospital Zurich, Zurich, Switzerland; 2 Division of Nephrology and Transplantation Medicine, HOCH Cantonal Hospital St.Gallen, St. Gallen, Switzerland; 3 Service of Nephrology, University Hospital Lausanne, Lausanne, Switzerland; 4 Division of Nephrology and Transplantation, University Hospital Geneva, Geneva, Switzerland; 5 Division of Nephrology, Cantonal Hospital Aarau, Aarau, Switzerland; 6 Department of Nephrology and Hypertension, Inselspital, Bern University Hospital, University of Bern, Bern, Switzerland; 7 Department of Urology, Inselspital, Bern University Hospital, University of Bern, Bern, Switzerland; 8 Department of Consultation—Liaison Psychiatry and Psychosomatic Medicine, University Hospital Zurich, University of Zurich, Zurich, Switzerland; 9 Institute of Diagnostic and Interventional Radiology, University Hospital Zurich, Zurich, Switzerland; 10 Institute of Physiology, University of Zurich, Zurich, Switzerland; 11 Institute for Nephrology and Dialysis, Cantonal Hospital Baden, Baden, Switzerland; Medical University of Vienna, AUSTRIA

## Abstract

**Introduction:**

Increasing use of low-dose abdominal computed tomography (CT) scans in clinical diagnostics and research offers high sensitivity for kidney stones with minimal radiation exposure. However, due to reduced specificity, incidental findings potentially lead to unnecessary follow-up, financial burden, and psychological distress. Gaps of knowledge remain regarding the prevalence of incidental findings and their financial and psychological consequences. This study investigates the prevalence of incidental findings in asymptomatic participants undergoing low-dose non-contrast CT scans and their economic and psychological sequelae.

**Methods:**

We conducted a retrospective, multicentric observational study using data from the assessment for the control group of the Swiss Kidney Stone Cohort (SKSC). Low-dose non-contrast CT scans were analyzed for incidental findings, cost and psychological impact. Statistical analyses evaluated participant characteristics, financial and psychological consequences.

**Results:**

229 participants underwent low-dose non-contrast CT scans, with 112 correctly completing the psychological questionnaires. The mean age was 42.9 years, and 56.3% were male. Incidental findings were observed in 47.2% (n = 108) of participants, with 16.6% having multiple findings. Kidney-related findings were the most prevalent, accounting for 35% of all findings. Of the incidental findings, 37.9% were classified as “incidentalomas” by the American College of Radiology (ACR) guidelines, and 15.7% of participants had findings that warranted follow-up according to radiology reports. In terms of costs, follow-up procedures, including imaging, consultations, and surgeries, incurred a total expense of 44’988 CHF, averaging 1967 CHF per participant and 2’999 CHF per incidental finding requiring follow-up. Surgical interventions were necessary for three participants, with individual costs reaching up to 35’208 CHF. Psychological assessment revealed that emotional distress and level of concern significantly differed across resilience levels and categories of CT findings. Participants with high resilience demonstrated lower emotional distress and concern, while those with CT findings requiring follow-up exhibited higher distress. Emotional distress was significantly greater in participants with follow-up findings compared to those without.

**Conclusions:**

Low-dose non-contrast abdominal CT scans often reveal incidental findings. Follow-up tests and procedures incurred significant financial costs, occasionally even leading to unnecessary surgical or non-surgical interventions. Psychological assessments showed increased anxiety in participants requiring follow-up, particularly those with low resilience. Our findings highlight the need for improved management, patient information, and consideration of economic and psychological impacts of incidental findings in clinical research and routine in the future.

## Introduction

The use of sectional imaging techniques is increasing, including low-dose computed tomography (CT) of the abdomen in the context of suspected nephrolithiasis [1]. CT scans, particularly low-dose, non-contrast types, are indispensable in modern diagnostics of kidney stones, balancing detailed imaging with minimized radiation exposure. Low-dose CT scans enable the detection of kidney calcifications or kidney stones with a sensitivity of nearly 98% and specificity of 97% [2]. Radiation exposure is low, ranging from 0.5–1.9 mSv. However, these scans also reveal incidental findings and the nature of low-dose, non-contrast imaging with limited spatial resolution can make it challenging to differentiate findings conclusively. Further work-up may be required to characterize incidental findings which increases the risk of unnecessary follow-up examinations and interventions [3,4]. This does not only generate costs but also harbors the risk of physical harm and health concerns. As such, the discovery of incidental findings can significantly impact participants’ psychological well-being [5]. While some individuals demonstrate resilience and adapt to the uncertainty or potential health implications, others experience anxiety and stress [6,7]. Resilience, or the ability to adapt to or recover from stress, may play a critical role in how participants cope with incidental findings.

Despite the growing body of literature on incidental findings, critical knowledge gaps remain, particularly regarding the prevalence of incidental findings in low-dose abdominal CT scans, a comprehensive analysis of their financial impact and their psychological implications. While a number of studies have assessed prevalence and clinical outcomes of incidental findings of other imaging modalities [8], fewer have examined the economic impact of these findings and, to our knowledge, no study has systematically examined the effect of incidental findings on psychological well-being [9]. In order to be able to weigh risks and benefits of an imaging test, clinicians need to know not only the risk of an imaging procedure itself but also the potential consequences of incidental findings. This is even more important when such examinations are conducted for scientific purposes on individuals who are asymptomatic.

This study aims to address these questions by investigating the prevalence and associated financial and psychological impact of incidental findings on abdominal low-dose non-contrast CT scans in asymptomatic participants.

## Methods and study population

### Study design

We conducted a retrospective, multicentric observational study using data from the screening for the control group of the Swiss Kidney Stone Cohort (SKSC).

The SKSC has been registered on ClinicalTrials.gov (NCT01990027) and was performed according to the Declaration of Helsinki, ICH-GCP, GEP, and Swiss law on human studies. The SKSC has been approved by the Swiss Cantonal Ethics Committees of all centers (KEK-ZH-Nr. 2013−0330; BASEC: PB_2016–01578) and encompasses participants with kidney stones and control subjects without history of urolithiasis and proven by abdominal CT to be free of kidney stones. Control patients signed an informed consent form that had been approved by the Swiss Cantonal Ethics Committees. Collection of demographic and clinical data and low-dose non-contrast CT scans were performed from 01.12.2017 to 11.03. 2020. The detailed protocol has been published elsewhere [10].

The current study was approved by the Swiss Cantonal Ethics Committees from all centers (Lausanne, Aarau, Geneva, Zurich; BASEC Nr. 2021−0109). All participants provided written informed consent. The data cannot be made publicly accessible for ethical and legal reasons. The data contains potentially identifying and sensitive information on the human study subjects. Requests from researchers who meet the criteria for access to confidential data can be submitted to the responsible Cantonal Ethics Committee Zurich (info.kek@kek.zh.ch).

Questionnaires for psychological assessment of resilience, emotional distress and level of concern were distributed (01.04.2023) and collected by mail (until 01.10.2023) from each center after the participants received their CT-scan results. Data from follow-up examinations related to the incidental findings, including clinical evaluations, laboratory tests, imaging studies, and interventions, were collected until August 2023. The data was accessed on 03.10.2023 and analysis of the coded data was performed centrally in Zurich.

### Study population

We investigated all individuals that were screened for the control group of the SKSC by four study centers across Switzerland (University Hospitals of Geneva, Lausanne and Zurich and the Cantonal Hospital Aarau) by abdominal CT scan. Distribution of participants across study centers is listed in S1 Table. For eligibility, subjects were 18 years or older, non-pregnant, and had signed a written informed consent. In this study, we included individuals with incidental kidney stones or calcifications on low-dose non-contrast CT of the screening visit who were excluded from the SKSC control group.

### Objectives and data collection

#### Imaging, demographic, clinical and laboratory data.

The analysis of the prevalence of incidental findings was conducted using images from low-dose non-contrast abdominal CT scans, as performed routinely, following a standardized CT protocol. Participants were scanned in a single breath-hold from the base of the lungs to 2–3 cm below the lower poles of the kidneys, without the use of contrast media. Incidental findings were classified according to the ACR guidelines on the management of incidental findings on abdominal CT [3]. Findings were independently assessed by radiology specialists experienced in abdominal CT interpretation. They were blinded to the study’s objectives ensuring an unbiased assessment of incidental findings. A total of 71 board-certified radiologists (attending level in tertiary care centers) independently assessed the CT scans. Approximately one-fifth of all scans (n = 54) were reviewed by the same radiologist, while nearly half were assessed individually by separate radiologists. This distribution reflects common clinical practice, with case allocation based on radiologist expertise, availability and institutional workflows. Demographic and clinical data (education level, smoking history, BMI, office blood pressure measurement) and laboratory values (total cholesterol, HbA1c, serum creatinine, and estimated glomerular filtration rate (eGFR)) from SKSC control subjects were obtained after the CT scan.

#### Cost analysis.

The cost analysis considered the expenses of additional tests (such as imaging and laboratory), treatments and hospitalizations as consequence of the incidental findings. The cost analysis was conducted from the healthcare system perspective, considering direct medical costs associated with additional tests, interventions, and follow-up procedures. The cost analysis included all direct medical costs incurred from the time of the initial CT scan until the final diagnosis and completion of primary work-up/treatment, where applicable. For this purpose, data was collected from the clinical information systems of the different study centers and billing information was gathered where available. Indirect costs such as loss of working hours of the participants were not taken into account. Costs are reported in Swiss Francs (CHF), with conversions to US Dollars (USD) based on the average exchange rate in 2023 (1 CHF = 1.13 USD).

#### Psychological evaluation.

The psychological evaluation consisted of two parts: first, assessment of resilience using the 6-item Brief Resilience Scale (BRS) [6], and second, assessment of the subjective psychological experience. For the BRS, responses to six questions were averaged to a score on a scale from 1 to 5 (low to high resilience). Based on the BRS, participants were categorized into groups with low (score 1.0 – < 3.0), normal (3.0–4.3), and high resilience (>4.3–5.0). To capture the subjective psychological experience of participants following the receipt of their CT scan findings, we used two modified questions from the Brief Illness Perception Questionnaire [11], which assessed emotional response (distress) and level of concern due to the CT scan results on a 10-point scale. Participants answered the questions, ‘How much did the results of your CT examination affect you emotionally on a scale from 1-10? (e.g., experiencing feelings of fear, agitation, or depression?)’ for emotional distress, and ‘How worried were you about receiving the results of your CT examination on a scale from 1-10?’ for level of concern, with higher scores indicating greater emotional distress or level of concern, respectively.

### Blinding

Participants were assigned coded SKSC IDs in a central, electronic database (SLIMS, Agilent, Santa Clara, CA, USA), which were also placed on the CT reports and questionnaires by the local study teams. Study subjects that were not part of the SKSC control group, because of kidney stones or calcifications identified in the screening CT, were also given a code, with the key only known to the local study team. The central investigators and radiologists of this study were unaware of the participants’ identities and had no access to information that could identify individual patients. The coded questionnaires were distributed and collected by the individual centers but were centrally analyzed in Zurich.

### Statistical analyses

The prevalence of incidental findings on low-dose non-contrast CT scans, was analyzed descriptively. Participant characteristics, including demographic data, clinical, and laboratory values, were summarized using means ± standard deviations for continuous variables and percentages for categorical variables. Differences between groups (participants with vs. without incidental findings) were assessed using unpaired t-tests for continuous variables and Chi-square tests for categorical variables. The Kruskal-Wallis test was utilized to evaluate differences in emotional distress and levels of concern across different resilience groups and categories of CT scan findings. The total and average costs of additional tests and treatments resulting from these findings were calculated and reported in Swiss Francs (CHF) as total cost and as average cost per CT-scan of our study. A p-value of <0.05 was considered statistically significant where applicable. Statistical analyses were performed using GraphPad Prism Version 10.0.0 for Windows (GraphPad Software, Boston, Massachusetts, USA, www.graphpad.com).

## Results

**Fig 1:**
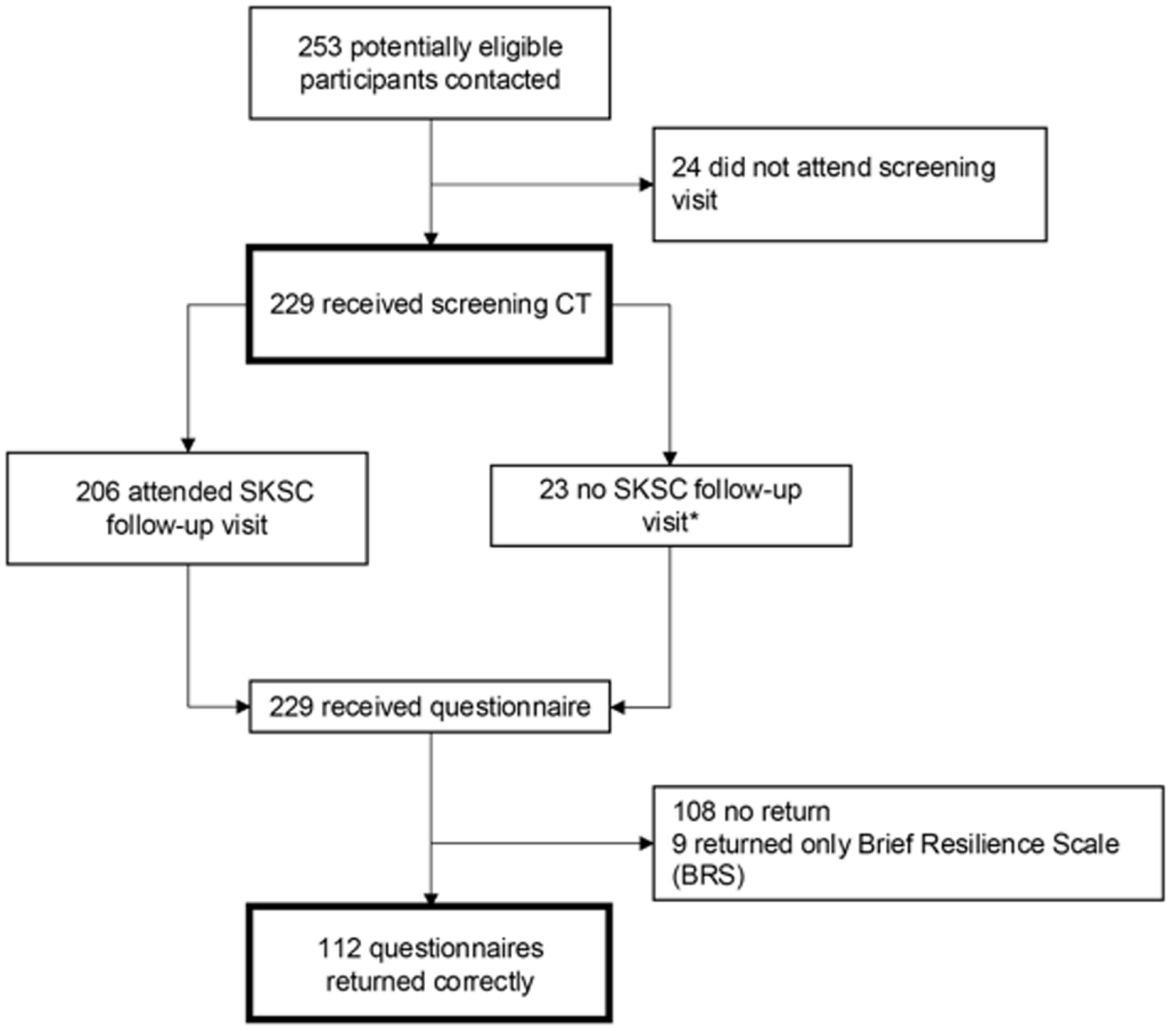
Study flowchart. *Participants (n = 23) with incidental urolithiasis/calcifications (n = 17) or failed attendance of Swiss Kidney Stone Cohort (SKSC) study visit (n = 6) that were excluded from the SKSC control group were retained in this study for further analysis of incidental computed tomography (CT) findings and psychological assessment.

Overall, 229 participants with CT scan were included in the study of 253 potentially eligible individuals. 112 study subjects returned the questionnaires correctly (Fig 1).

The mean age of all study participants was 42.9 (±13.4) years, with 56.3% being male. Study subjects with incidental findings on CT scan were significantly older, more often male and had significantly higher systolic blood pressure, glycated hemoglobin A1c (HbA1c) and serum creatinine resulting in lower eGFR compared to participants free of incidental findings as shown in Table 1. History of smoking, hypertension, diabetes and dyslipidemia as well as mean body mass index (BMI) did not differ significantly between groups.

**Table 1:** Characteristics of the study population.

	Incidental findings		
	No (N = 121)	Yes (N = 108)	p-value
age, years, mean (±SD)	38.5 (±10.9)	47.8 (±14.2)	p = 0.006*10^−5^
gender (male), n (%)	60 (49.6%)	69 (63.9%)	P = 0.029
European background, n (%)	117 (96.7%)	104 (96.3%)	P = 0.87
hypertension history, n (%)	7 (5.8%)	12 (11.1%)	P = 0.077
office systolic BPM, mmHg, mean (±SD)	117.3 (±13.2)	125.4 (±16.8)	P = 0.0001
office diastolic BPM, mmHg, mean (±SD)	73.3 (±11.4)	76.2 (±10.7)	P = 0.062
diabetes history, n (%)	1 (0.9%)	2 (1.8%)	P = 0.43
HbA1c, %, mean (±SD)	5.28 (±0.38)	5.44 (±0.73)	P = 0.041
dyslipidemia history, n (%)	14 (11.6%)	10 (9.3%)	P = 0.670
total cholesterol, mmol/L, mean (±SD)	4.7 (±0.96)	4.9 (±1.1)	P = 0.87
smoking history, n (%)	48 (39.7%)	31 (28.7%)	P = 0.094
serum creatinine, µmol/L, mean (±SD)	74.2 (± 13)	78.7 (±14.4)	P = 0.02
eGFR, mL/min/1.73m^2^, mean (±SD)	94 (± 9)	90 (± 13)	P = 0.007
BMI, kg/m^2^, mean (±SD)	24.6 (±4.3)	25.7 (±4.6)	P = 0.093
level of education, n (%)			P = 0.939
low	18 (14.9%)	16 (14.8%)	
middle	11 (9.1%)	8 (7.4%)	
high	74 (61.2%)	60 (55.5%)	

SD, Standard Deviation; BMI, Body Mass Index; BPM, Blood Pressure Measurement (mean of the 2nd and 3rd measurement out of three office measurements); HbA1c, glycated hemoglobin A1c; eGFR, estimated glomerular filtration rate (CKD-EPI 2009 formula) [12].

### Incidental findings

153 individual findings were identified in 47.2% (n = 108) of the 229 participants. 16.6% (n = 38) of the participants had more than one finding. 37.9% (n = 58) of the findings were classified as “incidentalomas” according to the ACR findings committee as shown in Table 2. 15.7% (n = 36) of the participants had a finding (classified by ACR or not) that warranted further follow-up according to radiology reports. Excluding kidney stones, 8.3% (n = 19) of participants required further follow-up. The kidneys accounted for 35% (n = 54) of all incidental findings. Notably, among the findings classified by ACR, there was a discrepancy between the follow-ups recommended by the ACR guidelines (9 cases) and those recommended by the radiology report (14 cases).

**Table 2:** Incidental findings categorized as incidentaloma by the American College of Radiology (ACR) Incidental Findings Committee.

Category, % (n)	Incidental findings (n = 153)	Participants (n = 229)	Participants requiring follow-up^1^ (n = 36)
findings	100.0% (153)	47.2% (108)	15.7% (36)
≥2 findings per participant	56.2% (86)	16.6% (38)	3.9% (9)
findings categorized by ACR	37.9% (58)	20.9% (48)	6.1% (14)
renal lesions	19% (29)	12.7% (29)	3.1% (7)
renal cysts	17.6% (27)	11.8% (27)	2.2% (5)
Bosniak I	15% (23)	10% (23)	0.4% (1)
Bosniak II	2% (3)	1.3% (3)	1.3% (3)
Bosniak IIF^*^	0.65% (1)	0.4% (1)	0.4% (1)
Bosniak III/IV^*^	0% (0)	0% (0)	0% (0)
solid mass <1 cm^*^	0% (0)	0% (0)	0% (0)
solid mass ≥1 cm^*^	1.3% (2)	0.9% (2)	0.9% (2)
liver lesions total	15.7% (24)	10.5% (23)	2.2% (5)
benign imaging features^2^	12.4% (19)	8.3% (19)	0% (0)
**suspicious imaging features**^3^^*^	3.3% (5)	2.2% (5)	2.2% (5)
adrenal lesions total	3.3% (5)	2.2% (5)	0.9% (2)
≤10 HU	2.6% (4)	1.7% (4)	0.4% (1)
>10 HU^*^	0.65% (1)	0.4% (1)	0.4% (1)

* (Bold) Findings warranting additional follow-up according to ACR guidelines;

^1^according to the radiology report;

^2^typical hemangioma, sharply marginated, homogeneous low attenuation (up to 20 HU), no enhancement, may have sharp, but irregular margins;

^3^ill-defined margins, enhancement (more than 20 HU), heterogeneous, enlargement; HU = Hounsfield Unit.

Incidental findings not included in the ACR White Paper were also tabulated as shown in Table 3. We identified 95 additional incidental lesions across various organ systems, with 22 cases necessitating further follow-up (17 urolithiasis and 5 other findings). Renal findings were most prevalent, comprising 25 instances, predominantly urolithiasis. All cases of incidental urolithiasis were classified as findings requiring follow-up due to the need for urinary and blood analysis as part of standard stone metaphylaxis.

**Table 3:** Incidental findings described by the radiologist but not categorized as incidentaloma by the American College of Radiology (ACR) Incidental Findings Committee.

Category, % (n)	Incidental findings (n = 153)	Participants (n = 229)	Participants requiring follow-up
findings not categorized by ACR	62.1% (95)	26.2% (60)	9.6% (22)
renal findings (n = 25)	16.3% (25)	10.9% (25)	7.4% (17)
liver lesions (n = 11)	7.2% (11)	4.8% (11)	0.4% (1)
adrenal lesions (n = 1)	0.7% (1)	0.4% (1)	0% (0)
pancreatic findings (n = 2)	1.3% (2)	0.9% (2)	0% (0)
spleen (n = 6)	3.9% (6)	2.6% (6)	0% (0)
chest findings (n = 16)	10.5% (16)	7% (16)	0.4% (1)
vascular findings (n = 12)	7.8% (12)	5.2% (12)	0% (0)
gastrointestinal tract (n = 7)	4.6% (7)	3.1% (7)	0.4% (1)
hernias (n = 7)	4.6% (7)	3.1% (7)	0% (0)
gender-specific findings (n = 4)	2% (3)	1.3% (3)	0.4% (1)
musculoskeletal findings (n = 9)	5.9% (9)	3.9% (9)	0% (0)
skin (n = 2)	1.3% (2)	0.9% (2)	0.4% (1)

### Cost analysis

Of the 36 participants identified by the radiology report to require follow-up, we have data showing that 15 subjects underwent the recommended follow-up procedures, including laboratory tests, imaging, outpatient consultations, and interventions. Among these, 3 participants required surgical interventions for their incidental findings.

The total financial cost for the additional tests and treatments associated with these incidental findings amounted to 44’988 CHF (approx. 50’836 USD). On a per-imaging-test basis, these additional treatments resulted in an average cost of 197 CHF (approx. 222 USD) per CT scan of subjects included in our study and 2’999 CHF (approx. 3389 USD) per incidental finding requiring follow-up. The median cost for follow-up was 771 CHF (approx. 871 USD). The individual costs and subsequent visits varied substantially among the 15 participants, ranging from as low as 99 CHF (111 USD) for a simple laboratory follow-up evaluation to as high as 35’208 CHF (39’774 USD) for multiple imaging follow-ups, hospital stays, and surgery of the incidental liver lesion in a single participant.

A total of 8 laboratory tests were conducted, accruing a cost of 1’194 CHF (1’349 USD). For imaging procedures, 20 follow up examinations were recorded, summing up to a cost of 5’762 CHF (6’517 USD). They consisted of 9 abdominal magnetic resonance imaging (MRI) scans, 7 abdominal ultrasounds, and 4 abdominal CT scans with contrast accounting for approximately 15 mSv per CT scan for these 4 individuals in addition to the approximately 1.5 mSv of the initial low-dose scan [13]. Regarding outpatient consultations, a total of 28 visits were recorded, with total expenses amounting to 3’311 CHF (3’746 USD). The majority of participants (53%) required only a single follow-up consultation. Additionally, 4 interventions were carried out, 1 laparoscopic removal of a liver lesion revealing a benign histologic lesion (hemangioma), 1 upper endoscopy showing no worrisome abnormalities and 2 ureterorenoscopies (URS) successfully removing the identified ureteral stones in 2 out of 5 participants with available follow-up data on kidney stones, amounting to a total of 34’721 CHF (39’242 USD). Direct costs are listed in Table 4. Ultimately, in the 15 participants for whom follow-up data were available, all follow-up procedures clarified the nature of the findings as benign, with only ureterorenoscopy providing a clear direct therapeutic benefit in 2 participants.

**Table 4:** Follow up costs for incidental findings.

Finding	Number of participants (n)	Total cost (CHF)	Average cost per lesion (CHF) (range)	Median number of visits (range)	Total interventions (n)
kidney stones	5	4’627	925 (99–2438)	1 (1–7)	2
hypodense liver lesion	4	36’182	9045 (276–35’207)	2 (1–9)	1
renal cyst Bosniak II/IIF	3	905	302 (233–348)	1	0
adrenal adenoma	1	1’669	1’669	3	0
solid renal mass >1 cm	1	771	771	3	0
thickened gastric wall	1	835	835	1	1

### Psychological evaluation

The questionnaire, used for assessment of resilience and subjective psychological experience, was completed by 112 participants. 12 subjects were classified into the low, 69 into the normal, and 31 into the high resilience group. We examined variations in emotional distress and level of concern, measured on a 10-point scale, across different resilience groups and categories of CT results (‘No finding’, ‘No follow-up’ and ‘Follow-up’). 9 participants, who only provided information for the BRS but no information about these aspects were excluded from this analysis.

**Fig 2:**
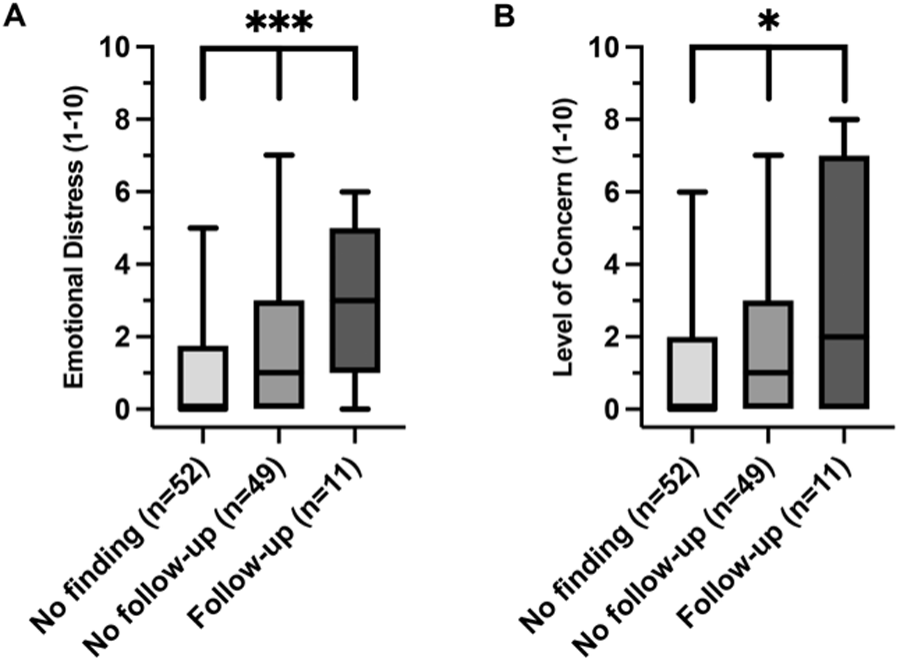
‘Emotional Distress’ (A) and ‘Level of Concern’ (B) across CT findings categories. Box plots illustrating the distributions of ‘Emotional Distress’ (A) and ‘Level of Concern’ (B) across CT findings categories: ‘No finding’, ‘No follow-up’ and ‘Follow-up’. Emotional distress was assessed with the question, “How much did the results of your CT examination affect you emotionally (scale 1-10) (e.g., having feelings of fear, agitation, or depression)?”, and level of concern was gauged by asking, “How worried were you about receiving the results of your CT examination (scale 1-10)?”. Statistical significance, as determined by Kruskal-Wallis test, is denoted above the relevant groups with *representing a p-value threshold of p < 0.05, **p ≤ 0.01 and ***p ≤ 0.001.

**Fig 3:**
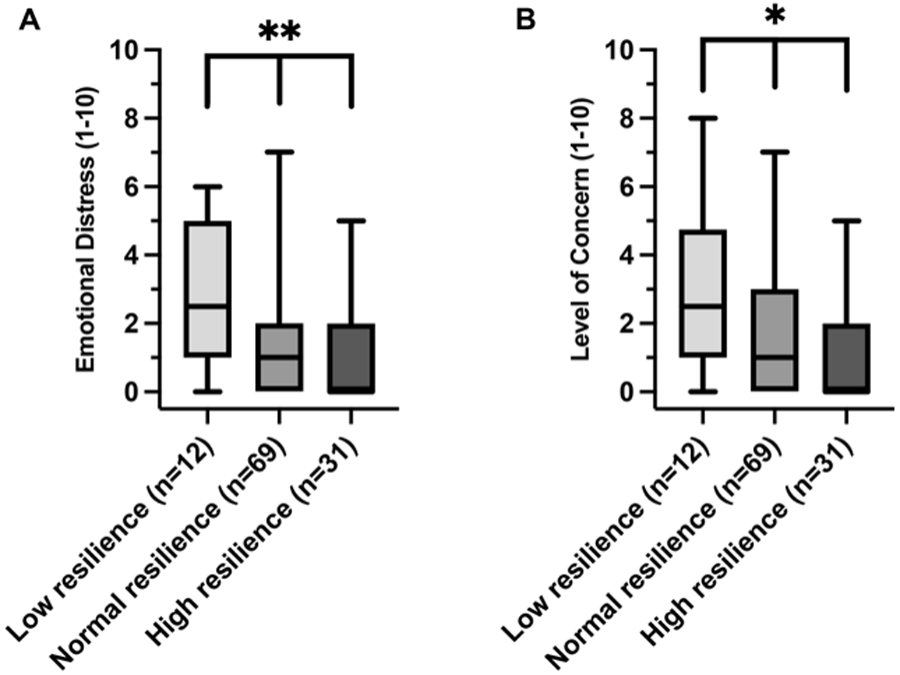
‘Emotional Distress’ (A) and ‘Level of Concern’ (B) across resilience groups. Box plots illustrating the distributions of ‘Emotional Distress’ (A) and ‘Level of Concern’ (B): for each resilience group (Low, Normal, High). Emotional distress was assessed with the question, “How much did the results of your CT examination affect you emotionally (scale 1-10) (e.g., having feelings of fear, agitation, or depression)?”, and level of concern was gauged by asking, “How worried were you about receiving the results of your CT examination (scale 1-10)?”. Statistical significance, as determined by Kruskal-Wallis tests, is denoted above the relevant groups with *representing a p-value threshold of p < 0.05, **p ≤ 0.01 and ***p ≤ 0.001.

Participants across the three categories of CT scan results showed significant differences in the median scores for emotional distress (p < 0.001) and level of concern (p = 0.03) as illustrated in Fig 2. Additionally, when analyzing by resilience group, both emotional distress and level of concern showed significant variances across resilience groups, with the high resilience group exhibiting the lowest median scores for emotional distress (p = 0.006) and level of concern (p = 0.016) as shown in Fig 3.

To explore the differences in emotional distress and level of concern further, a Dunn’s post hoc analysis was conducted. The post-hoc analysis revealed that participants with no CT findings had significantly lower emotional distress scores compared to those requiring follow-up (p = 0.0034) and those who did not require follow-up (p = 0.0096) (S3A Table). However, there were no statistically significant differences in the level of concern in the individual group comparisons of the finding categories (S3B Table). For the three resilience groups, a significant difference in emotional distress (p = 0.0050) and level of concern (p = 0.0141) was found between the low and high resilience groups, while the difference observed between other resilience groups were not statistically significant (S4A Table and S4B).

**Fig 4:**
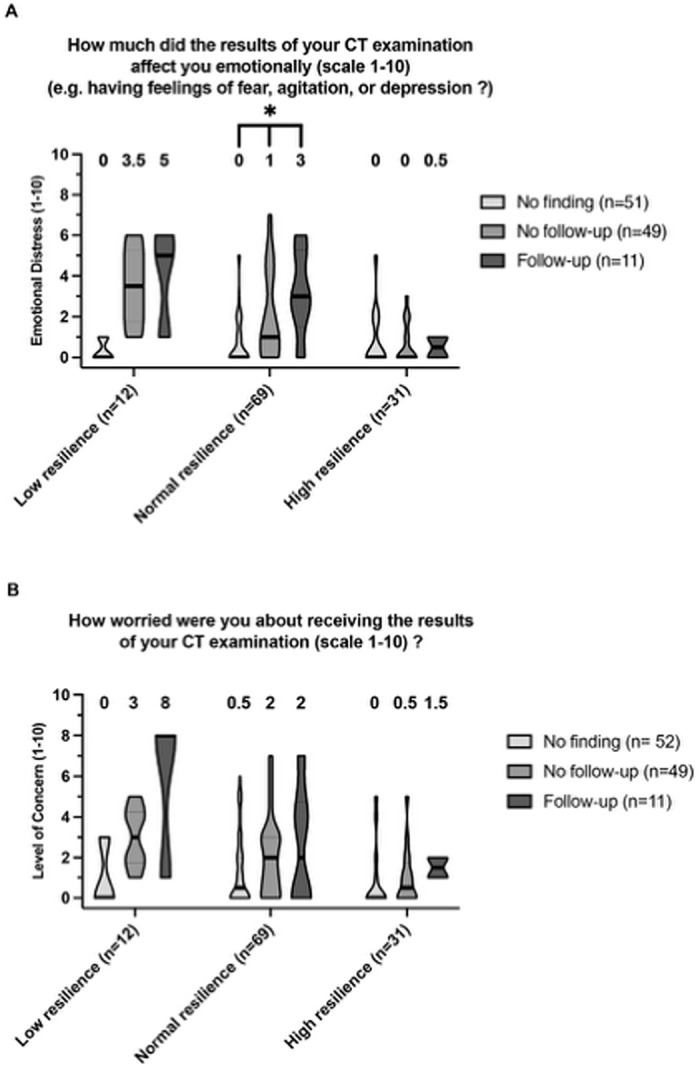
Distribution of ‘Emotional Distress’ (A) and ‘Level of Concern’ (B) across resilience groups. Box plots representing distributions of ‘Emotional Distress’ (A) and ‘Level of Concern’ (B) for each resilience group (Low, Normal, High) subdivided by CT findings (No finding, No follow-up, Follow-up). The boxes show the range from the maximum to the minimum value observed, with the median values indicated within (as horizontal lines) and above each box. Statistical significance, as determined by Kruskal-Wallis test, is denoted above the relevant groups with asterisks representing a p-value threshold of p < 0.05.

The subsequent stratification by resilience group (Fig 4) showed, that in the low resilience group, participants with ‘Follow-up’ findings displayed higher median scores in emotional distress and level of concern compared to ‘No finding’ or ‘No follow-up’ categories. However, these differences were not statistically significant for both emotional distress (p = 0.0574) and level of concern (p = 0.1674).

The normal resilience group demonstrated a moderate emotional distress, especially in the ‘Follow-up’ category (median emotional distress of 3), with a significant difference in emotional distress across finding categories (p = 0.0026), but not in the level of concern (p = 0.2).

The high resilience group reported uniformly low median scores in both emotional distress and level of concern across all finding categories, with no significant differences (p = 0.99 and p = 0.37 respectively).

## Discussion

Low-dose non-contrast abdominal CT is primarily used to diagnose or exclude urolithiasis. It only requires moderate doses of radiation and no intravenous (IV) contrast [14–16]. However, its lower spatial resolution and lack of contrast may lead to incidental findings that cannot be clearly assessed for their significance. We found a high prevalence of incidentalomas. There was a total of 153 incidental findings in 47% of the 229 participants. Most other studies in the literature examining incidental findings in *unenhanced* abdominal CT have focused on cohorts of patients presenting with symptoms indicative of nephro- or urolithiasis, such as flank pain or other signs of urinary colic [17–19]. Consequently, these cohorts are not directly comparable to ours with asymptomatic subjects. A recent meta-analysis investigated the frequency of extracolonic incidentalomas in CT colonoscopy, an abdominal imaging modality which, like low-dose non-contrast abdominal CT, usually does not involve intravenous contrast media and is used in individuals which are mostly asymptomatic [8,20]. The authors found an average frequency of extracolonic incidentalomas of 38% (range 21–57%) which is roughly comparable to the incidence in our study. However, 3 of the 11 primary studies included in the meta-analysis used IV contrast. Another study investigated incidental findings in low-dose unenhanced abdominal CT followed by a combined arterial and urographic phase in asymptomatic potential kidney donors [21]. Out of 375 participants in the study, 158 individuals (42%) had incidental findings. Among these 158 participants, 228 incidental findings were discovered. However, only 7.5% had incidental findings requiring follow up [21].

In our study, recommendations for follow-up were made by the radiologist. 15.7% of the study subjects required follow-up for incidentalomas. Follow-up in our study was more frequent compared to numbers reported for similar imaging modalities using higher radiation doses and/or contrast in asymptomatic or symptomatic individuals ranging from 3.5% to 11.7% [21–26]. The reason for the higher rate of follow-ups in our study participants may be attributed to the shortcomings of low-dose non-contrast abdominal CT. Minimizing radiation exposure reduces imaging quality and impairs the ability to accurately distinguish findings. This may lead to a conservative approach, favoring follow-up to ensure participant safety and diagnostic accuracy. Of course, the higher rate of follow-ups compared to data in the literature could be explained by other factors, such as differences in the characteristics of the cohort, particularities of the respective health system, guidelines on the management of incidental findings, etc.

While some participants in our study benefited from the discovery of findings, this was not the case for others. The frequency of follow-up investigations matters, because they may not only detect relevant pathologies but also potentially increase the risk of adverse outcomes. Ensuing harm may be of physical, economic or psychological nature. In our study, 4 of the 112 participants had to undergo a follow up high-resolution contrast CT scan imposing an additional radiation dose of 15 mSv per individual. Of the 36 participants in our study who were recommended follow-up, 33 had benign follow-up findings, which did not require further measures. However, 3 of 36 subjects underwent a surgical intervention for their incidental findings. 2 individuals received ureterorenoscopic extraction of a calculus, which was deemed too large for spontaneous passage. The third participant underwent a partial hepatectomy, which ultimately revealed a benign histologic lesion (hemangioma). Stone extraction potentially benefitted the individuals. The third study participant, however, did not benefit from liver surgery in the end, even though it was appropriate given the inability of subsequent imaging to definitively determine the lesion’s nature. Ultimately, this study participant underwent an intervention that exposed him to substantial risks and incurred significant follow-up costs. This case effectively underscores the direct potential health risks of incidental imaging findings, which are even more concerning when the initial examination was performed for research purposes rather than a medical necessity.

In addition to physical harm, there are economic consequences of incidental findings. Our study demonstrates relevant financial costs of follow-up tests and treatments. There were total follow-up costs of 44’988 CHF (50’836 USD) equaling 197 CHF (222 USD) per initial low-dose abdominal CT. This corresponds to an additional 109% on top of the costs for the abdominal CT in Switzerland (180 CHF; 203 USD). As we did not have follow-up information of all participants, costs may have been even higher. Additionally, indirect costs resulting from loss of working days, which were not assessed in this study, would further add to the economical consequences. Previous studies across imaging modalities, including cardiac CT and MR imaging, have shown that incidental findings frequently lead to additional diagnostic procedures, follow-up imaging, and interventions.[27–35]. However, no study so far has approximated follow-up costs in a reasonable granularity as we did. Taken together, our findings underscore the importance of precisely determining the indication for such an examination, as any expansion of the indication criteria may lead to significantly higher subsequent costs. This is supported by the literature, which suggests that reducing unnecessary follow-up can effectively reduce costs while maintaining high standards of patient care [3]. Advanced technologies, including artificial intelligence (AI), have the potential to enhance the differentiation between clinically significant findings and benign incidentalomas, thereby reducing the need for follow-up procedures and associated costs in the future [36]. Based on these observations, our study elucidates the significant financial burden of incidental findings.

The third dimension of harm that may be caused by incidental findings is psychological distress. We show that emotional distress and level of concern was significantly associated with the finding category (‘No finding’, ‘No follow-up’ and ‘Follow up’). Notably, significant differences in emotional distress and levels of concern were observed across the ‘No finding’, ‘No follow-up’ and ‘Follow-up’ categories, indicating that the necessity for follow-up procedures significantly heightens participants’ anxiety and worry.

With regard to the psychological impact of incidental findings, the degree of emotional distress and concern seemed to be dependent on individual resilience. The participants with the highest resilience demonstrated the lowest median score for emotional distress and level of concern. Our study population showed a mean BRS score of 3.8, which is comparable to the population of the validation studies for the French and German BRS (mean BRS scores of 3.5 and 3.7, respectively) [37,38]. This underlines the generalizability of our cohort and excludes that we had a bias in this group of individuals. It could have been speculated that primarily anxious subjects chose to participate in this study, seeking to rule out intra-abdominal pathologies through the CT scan free of cost. Conversely, it could also have been possible that there was a bias towards individuals with high levels of resilience because they were undeterred by the radiation or the blood tests.

Interestingly, out of the 112 participants who completed the psychological questionnaires, 11 had undergone follow-up procedures. This represents a significant proportion (73.3%) of the 15 participants who required follow-up even though only 112/229 (49.0%) of the control subjects returned the entire questionnaires. The high percentage of participants who required follow-up procedures and participated in our survey may be linked to a greater emotional burden they experienced. This emotional strain could have increased their motivation or perceived need to reflect on and express their psychological well-being via the questionnaire.

The results of the stratification of participants by resilience highlights the potential influence of psychological resilience on how individuals cope with incidental findings, as suggested by the observed differences in emotional distress and level of concern across resilience groups. The limited sample sizes, especially in the low and high resilience groups, may have prevented the observed differences from reaching statistical significance, though the trend remains suggestive. Previous research in whole-body MRI screening has shown that the psychological impact of incidental findings can persist long after disclosure, with distress often disproportionate to the clinical significance of the findings [39–42]. Our data not only support these observations but also emphasize the moderating role of psychological resilience, underscoring its potential relevance in pre-screening assessments and post-disclosure counseling.

Our study highlights the complex and far-reaching impact that incidental findings can have on individuals. These situations may arise in two distinct contexts: clinical diagnostics and research or study settings.

In *routine* diagnostics, it should be mandatory that patients receive thorough information about all aspects of the examination prior to undergoing radiologic examinations. Oral information by the physician and written informed consent forms should not only encompass the risks associated with CT scans, such as increased radiation exposure and allergic or adverse renal reactions to contrast agents, as is currently the case. They should also stress the likelihood and potential consequences of significant incidental findings and that such findings may lead to additional physical risks, including further examinations with substantial radiation exposure or even surgical interventions. Moreover, it should be conveyed that incidental findings can result in significant additional costs, which may sometimes fall on the patient. Lastly, it needs to be mentioned that incidental findings can trigger psychological and emotional distress, particularly when their nature is unclear or remains unresolved over time. Such demands have been made in earlier publications, however, at least in Switzerland, they have not been incorporated into clinical practice [43].

Many clinical trials or prospective studies today utilize radiological imaging that is not essential for study subjects in either the control or the treatment groups [10,44]. Yet, guidelines governing the use of research imaging are often inconsistent and limited, failing to account fully for the interests of participants [45]. Improved standards for handling research images and incidental findings are urgently needed, particularly in studies where radiological examinations are not required for medical reasons. Unlike routine clinical diagnostics, participants in research studies do not directly benefit from the imaging performed. In this context, it is even more critical to implement comprehensive measures to protect individuals from any potential harm. Rigorous ethical considerations are necessary, ensuring thorough participant information. During the consent process, subjects must be informed not only of the potential benefits and risks of the test, but also of how images will be managed, whether incidental findings will be identified, how those findings will be disclosed, what further investigations will be pursued, and the possible physical, financial, and psychological implications. Regarding the costs of follow-up investigations or treatments, it may be worth discussing whether these should be covered by the study insurance. Our data could aid in better estimating these associated risks. To address the psychological effects of incidental findings, offering psychological support to participants in the event of such discoveries, could also be considered. Another approach how to mitigate psychological distress may involve assessment of the resilience of participants during the screening period and prior to enrollment. BRS can be assessed by only 6 questions, which would allow for a relatively easy implementation not only in scientific studies but also in clinical routine. Individuals with high resilience in our study demonstrated lower median scores for both emotional distress and level of concern, suggesting a robust capacity to manage stress associated with incidental CT findings compared to low resilience individuals. It would be conceivable to exclude individuals with low resilience scores from studies to minimize the risk of psychological harm. Naturally, this strategy would need to be applied consistently across both control and study groups to avoid introducing bias. It would also make it more difficult to recruit enough study participants in clinical trials. If the exclusion of patients with low resilience is not desirable, resilience data could at least be leveraged to tailor communication and management strategies to individual needs. Overall, future research should further explore in a prospective manner on how to best deal with these issues, aiming to enhance patient care while maintaining economic sustainability.

Our study has several strengths. One is the multicentric approach, which allows for better generalizability. Other novelties are the integration of follow up costs in high granularity and the quantification of the psychological impact. Aside from the limited number of participants in certain subgroups, our study also has shortcomings. Cost analysis was probably not complete as we could only incorporate follow-up procedures that were known to the study centers. Extramural follow-up assessments, performed for example by primary care physicians, might not have been entirely captured and indirect costs resulting from loss of working hours have not been captured. Therefore, our study most likely underestimates follow up costs. A second limitation is the retrospective design, which, in combination with the reliance on self-reported data, might promote recall bias regarding the psychological impact. However, a positive aspect of the retrospective nature is that it captures the emotional impact, which has persisted in the participants’ memories. Therefore, recall bias in this case is not necessarily a limitation but could also be interpreted as representative of the memorized emotional distress, as it reflects enduring psychological harm that participants associate with their incidental findings. Prospective studies are needed to validate our findings and assess the short and long-term outcomes of different incidental findings management strategies.

## Conclusions

In conclusion, this is the first large cohort study that comprehensively investigates prevalence, psychological and financial consequences of incidental findings in non-contrast low-dose abdominal CTs in a well-characterized population of asymptomatic individuals. We demonstrate that the prevalence of incidental findings on low-dose non-contrast CT scans is substantial and follow-up examinations are frequent also in an asymptomatic population in a research setting. The associated costs must not be neglected and psychological impact of these findings varies widely and depends at least partially on the resilience of the study subject. In light of our findings, adopting a personalized approach in studies and clinical practice, which considers psychological resilience and optimizes management strategies for incidental findings to balance health implications, economic costs and psychological impact, appears reasonable. Future prospective studies are needed to further explore these implications. A stricter information policy regarding the potential consequences of incidental findings in the study setting and the coverage of follow up costs may need to be implemented.

## Supporting information

S1 Table Distribution of participants across study centers. Details the distribution of 299 participants across four major study centers, presenting the total number and percentage of participants at each location.
(DOCX)

S2 Table Incidental findings not classified by ACR. A more detailed enumeration of incidental findings not categorized by the American College of Radiology (ACR), listing categories, subcategories, and details such as percentages and participant counts. It also highlights individual findings that warrant additional follow-up marked with an asterisk.
(DOCX)

S3a Table Results of Dunn’s post hoc analysis following Kruskal-Wallis test for emotional distress across finding categories. Detailed analysis of emotional distress across various finding categories, showcasing median group comparisons, adjusted p-values, and significance annotations, where non-significant results are indicated as ‘ns’.
(DOCX)

S3b Table Results of Dunn’s post hoc analysis following Kruskal-Wallis test for level of concern across finding categories. Detailed analysis of levels of concern across various finding categories, showcasing median group comparisons, adjusted p-values, and significance annotations, where non-significant results are indicated as ‘ns’.
(DOCX)

S4a Table Results of Dunn’s post hoc analysis following Kruskal-Wallis test for emotional distress across resilience groups. Detailed analysis of emotional distress across different resilience groups, showcasing median group comparisons, adjusted p-values, and significance annotations, where non-significant results are indicated as ‘ns’.
(DOCX)

S4b Table Results of Dunn’s post hoc analysis following Kruskal-Wallis test for level of concern across resilience groups. Detailed analysis of level of concern across different resilience groups, showcasing median group comparisons, adjusted p-values, and significance annotations, where non-significant results are indicated as ‘ns’.
(DOCX)
