# Supplementary material for: Incidental findings on non-contrast abdominal computed tomography in an asymptomatic population: Prevalence, economic and health implications
Source: PLoS One. 2025 Aug 4;20(8):e0328049. doi: 10.1371/journal.pone.0328049 (PMC12321107; doi:10.1371/journal.pone.0328049)
Supplement: S1 Table — Details the distribution of 299 participants across four major study centers, presenting the total number and percentage of participants at each location. (DOCX) [file pone.0328049.s001.docx]

**S1 Table:** Distribution of participants across study centers

| **Study center** | **Total participants (n = 299)** | **Percentage of total participants**  **(%)** |
| --- | --- | --- |
| University Hospital Zurich | 120 | 52.4% |
| University Hospital Geneva | 31 | 13.5% |
| University Hospital Lausanne | 73 | 31.9% |
| Cantonal Hospital Aarau | 5 | 2.2% |
