# Supplementary material for: Incidental findings on non-contrast abdominal computed tomography in an asymptomatic population: Prevalence, economic and health implications
Source: PLoS One. 2025 Aug 4;20(8):e0328049. doi: 10.1371/journal.pone.0328049 (PMC12321107; doi:10.1371/journal.pone.0328049)
Supplement: S2 Table — A more detailed enumeration of incidental findings not categorized by the American College of Radiology (ACR), listing categories, subcategories, and details such as percentages and participant counts. It also highlights individual findings that warrant additional follow-up marked with an asterisk. (DOCX) [file pone.0328049.s002.docx]

**S2 Table:** Incidental findings not classified by ACR

| **Category, % (n)** | **Subcategory** | **Details** | **Incidental findings**  **(n=153)** | **Participants (n=229)** | **Participants requiring**  **follow-up** |
| --- | --- | --- | --- | --- | --- |
| findings not  categorized by ACR |  |  | 62% (95) | 26.2% (60) | 9.6% (22) |
| renal findings  (n=25) | **urolithiasis*** |  | 11.1% (17) | 7.4% (17) | 7.4% (17) |
|  | anatomical anomalies | unilateral small kidney, horseshoe kidney, renal malrotation, ectasia of renal pelvis, calyceal  diverticulum | 3.9% (6) | 2.6% (6) | 0% (0) |

|  | other renal findings | unspecific calcification, unspecific  hyperdensity | 1.3% (2) | 0.9% (2) | 0% (0) |
| --- | --- | --- | --- | --- | --- |
| liver lesions  (n=11) | fatty liver and  calcifications |  | 3.9% (6) | 2.6% (6) | 0% (0) |
|  | cysts and stones | biliary cyst, cholecystolithi  asis | 2.6% (4) | 1.7% (4) | 0% (0) |
|  | infections | **cyst suspicious for echinococcus**  **infection*** | 0.65% (1) | 0.4% (1) | 0.4% (1) |
| adrenal lesions  (n=1) |  | adrenal  calcification | 0.65% (1) | 0.4% (1) | 0% (0) |
| pancreatic findings (n=2) |  | chronic pancreatitis, pancreatic  lipoma | 1.3% (2) | 0.9% (2) | 0% (0) |
| spleen (n=6) | enlargement  or cysts | splenomegaly,  splenic cyst | 3.3% (5) | 2.2% (5) | 0% (0) |
|  | tumors | splenic  hemangioma | 0.65% (1) | 0.4% (1) | 0% (0) |

| chest findings  (n=16) | pulmonary  nodules | < 6 mm | 1.3% (2) | 0.9% (2) | 0% (0) |
| --- | --- | --- | --- | --- | --- |
|  |  | **≥ 6mm*** | 0.65% (1) | 0.4% (1) | 0.4% (1) |
|  | inflammatory and other conditions | pulmonary granuloma, pulmonary  bullae | 5.9% (9) | 3.9% (9) | 0% (0) |
|  | congenital  anomalies | Bochdalek  hernia | 2.6% (4) | 1.7% (4) | 0% (0) |
| vascular findings (n=12) | aortic and arterial anomalies | small infrarenal aortic aneurysm, aneurysm of  splenic artery | 3.3% (5) | 2.2% (5) | 0% (0) |
|  | lymphatic system  abnormalities | insignificant lymphadenopa  thy | 3.3% (5) | 2.2% (5) | 0% (0) |
|  | other vascular  anomalies |  | 1.3% (2) | 0.9% (2) | 0% (0) |
| gastrointestinal  tract (n=7) | diverticulosis | diverticulosis | 3.9% (6) | 2.6% (6) | 0% (0) |
|  | wall  abnormalities | **gastric wall**  **thickening*** | 0.65% (1) | 0.4% (1) | 0.4% (1) |

| hernias (n=7) |  | umbilical,  inguinal, hiatal | 4.6% (7) | 3.1% (7) | 0% (0) |
| --- | --- | --- | --- | --- | --- |
| gender-specific findings (n=4) | female | ovarian cyst, **subcutaneous breast**  **nodule*** | 0.65% (1) | 0.4% (1) | 0.4% (1) |
|  | male | prostatic calcification, retractile  testes | 1.3% (2) | 0.9% (2) | 0% (0) |
| musculoskeletal findings (n=9) | degenerative changes | significant  vertebral osteoarthrosis | 3.3% (5) | 2.2% (5) | 0% (0) |
|  | congenital and developmental anomalies | benign osseous lesion, Schmorl's nodules,  scoliosis | 2% (3) | 1.3% (3) | 0% (0) |
|  | anatomic variants | vertebral anatomic  anomaly | 0.65% (1) | 0.4% (1) | 0% (0) |

| skin (n=2) | unspecified lesions | **unspecific skin lesion***, unspecific subcutaneous  calcification | 1.3% (2) | 0.9% (2) | 0.4% (1) |
| --- | --- | --- | --- | --- | --- |

*** (Bold) Findings warranting additional follow-up
