# Supplementary material for: Incidental findings on non-contrast abdominal computed tomography in an asymptomatic population: Prevalence, economic and health implications
Source: PLoS One. 2025 Aug 4;20(8):e0328049. doi: 10.1371/journal.pone.0328049 (PMC12321107; doi:10.1371/journal.pone.0328049)
Supplement: S3b Table — Detailed analysis of levels of concern across various finding categories, showcasing median group comparisons, adjusted p-values, and significance annotations, where non-significant results are indicated as ‘ns’. (DOCX) [file pone.0328049.s004.docx]

**S3b Table:** Results of Dunn’s post hoc analysis following Kruskal-Wallis test for level of concern across finding categories.

| **Comparison** | **Median Group 1** | **Median Group 2** | **Adjusted p- value** | **Significance** |
| --- | --- | --- | --- | --- |
| No Findings (n=52) vs. No  Follow-up (n=49) | 0 | 1 | 0.1134 | ns |
| No Findings (n=52) vs. Follow-  up (n=11) | 0 | 2 | 0.0886 | ns |
| No Follow-up (n=49) vs. Follow-up (n=11) | 1 | 2 | >0.9999 | ns |

Note: *p < 0.05, **p < 0.01, ***p < 0.001, ns = not significant.
