# Supplementary material for: Incidental findings on non-contrast abdominal computed tomography in an asymptomatic population: Prevalence, economic and health implications
Source: PLoS One. 2025 Aug 4;20(8):e0328049. doi: 10.1371/journal.pone.0328049 (PMC12321107; doi:10.1371/journal.pone.0328049)
Supplement: S4a Table — Detailed analysis of emotional distress across different resilience groups, showcasing median group comparisons, adjusted p-values, and significance annotations, where non-significant results are indicated as ‘ns’. (DOCX) [file pone.0328049.s005.docx]

**S4a Table:** Results of Dunn’s post hoc analysis following Kruskal-Wallis test for emotional distress across resilience groups.

| **Comparison** | **Median**  **Group 1** | **Median**  **Group 2** | **Adjusted p-**  **value** | **Significance** |
| --- | --- | --- | --- | --- |
| Low resilience (n=12) vs.  Normal resilience (n=69) | 2.5 | 1 | 0.1305 | ns |
| Low resilience (n=12) vs. High  resilience (n=31) | 2.5 | 0 | 0.0050 | ** |
| Normal resilience (n=69) vs.  High resilience (n=31) | 1 | 0 | 0.1280 | ns |

Note: *p < 0.05, **p < 0.01, ***p < 0.001, ns = not significant.
